# Advancing translational research in binge-eating: Integrating insights from clinical practice into animal models

**DOI:** 10.1038/s41398-026-04035-0

**Published:** 2026-04-11

**Authors:** Rachel Dufour, Uri Shalev, Linda Booij

**Affiliations:** 1 Department of Psychology, Concordia University, Montreal, QC Canada; 2 Research Centre, Douglas Mental Health University Institute, Montreal, QC Canada; 3 Eating Disorders Continuum, Douglas Mental Health University Institute, Montreal, QC Canada; 4 Center for Studies in Behavioral Neurobiology, Concordia University, Montreal, QC Canada; 5 Department of Psychiatry, McGill University, Montreal, QC Canada

**Keywords:** Human behaviour, Psychiatric disorders

## Abstract

Binge-eating behaviors are key components of several types of eating disorders, yet their etiology remains unclear. Animal models have provided valuable insights by enabling experimentally controlled investigations of biological, behavioral, and environmental factors contributing to eating disorders. This narrative review examines the clinical relevance of animal models in advancing our understanding of binge-eating-related disorders. We propose five translational priorities focused on clinically-meaningful features of binge eating and their relevance for improving animal-model development: (1) loss of control and compulsivity, (2) negative affect and stress responsivity, (3) developmental timing and sex differences, (4) individual differences and variability, and (5) treatment responsiveness. Various animal models, including food restriction, stress-induced, and addiction-based paradigms, have been developed to study binge eating. Limitations include the inability of animal models to fully capture the psychological and sociocultural dimensions of binge eating, such as the sense of loss of control, stigma, distress, and body-image concerns. While existing models capture key biological and behavioral components of binge eating, closer alignment with clinically defining features, for example, through the inclusion of emotional stressors and varied outcome measures, could improve translational impact. By refining current models to match clinical reality, animal research may continue to enhance our understanding of eating disorders and inform the development of novel treatment approaches.

## Introduction

In the last few decades, the prevalence of eating disorders (EDs), characterized by maladaptive eating behaviors such as extreme calorie restriction, binge-eating, and self-induced vomiting, has slowly risen, with lifetime prevalence reaching approximately 7.8% worldwide [1]. EDs can affect various facets of life, including general quality of life, academic pursuits, reproductive health, and social relationships [2]. Among these behaviors, binge eating is one of the most common clinical presentations, cutting across multiple ED diagnoses.

Binge-eating behaviors are associated with a range of both physiological and psychosocial consequences. Physiologically, they are linked to increased risk for type 2 diabetes and cardiovascular disorders [3]. Psychosocially, individuals may experience stigma, social isolation, and victimization [4]. Objective binge eating, defined as eating an objectively large amount of food in a short amount of time, while experiencing a sense of loss of control and intense distress, is part of the clinical presentation of the binge-eating disorder (BED) and bulimia nervosa (BN) [5]. These behaviors are also present in a subtype of anorexia nervosa and other specified feeding or eating disorders (OSFED). Individuals with EDs also could have subjective binges, in which the individual experiences loss of control and distress over a meal that other people would not consider excessively large [5]. In contrast, the definition of overeating is less widely accepted as a clinical symptom and lacks a formal operational definition, but is typically defined as eating more calories than one has expended or expelled, or eating outside of hunger [6].

The development of EDs is typically conceptualized through a biopsychosocial lens [4]. The biopsychosocial model emphasizes the interplay of genetics, brain processes, psychological factors, and adverse socioenvironmental influences. For example, for binge eating, heightened sensitivity to reward cues may interact with poor emotion regulation and a history of adverse experiences, contributing to loss-of-control eating in response to stressful events. In line with the biopsychosocial model, the cognitive-behavioral model for EDs highlights the central role of weight and shape overvaluation (i.e., the importance weight, shape, and eating have in determining an individual’s self-worth), dietary restraint, and negative emotions in the development and maintenance of symptoms [7]. In the case of binge eating, the cognitive-behavioral approach emphasizes a cycle in which strict dieting and negative emotions trigger binge episodes, which in turn reinforce distress and attempts to restrict and/or purge, perpetuating the disorder. Whereas social experiences and psychological factors are considered important determinants for ED risk and course, the role of biological factors in cognitive-emotional ED symptoms is not well understood.

Methodological limitations of human studies in the field of EDs contribute to difficulties in understanding these disorders better and refining their etiological and maintenance models. For example, clinical heterogeneity in symptom presentation and the presence of psychological and physical comorbidities contribute to increased variability in research data, which, on the other hand, reflects the realities of clinical practice. Furthermore, as adverse experiences cannot be ethically randomized in human studies, human binge-eating models cannot disentangle genetic factors from environmental influences when investigating binge-eating etiology [8]. Animal models were developed to study key aspects of EDs that are difficult to isolate and modify in a highly controlled environment in humans.

### Aims and objectives

Animal models have been central to elucidating the neurobiological and behavioral mechanisms underlying binge eating. However, translational progress has been limited by variability in how clinically-relevant features of binge eating are operationalized across animal models. The present review is intended for both preclinical researchers seeking to refine animal models and clinically-oriented researchers aiming to better interpret animal findings. We propose five translational priorities focused on clinically-meaningful features of binge eating and their relevance for improving animal-model development. After a brief overview of commonly-used animal models, we outline these priorities and provide strategic directions to guide future animal research.

## Animal models of binge eating and overeating

The models reviewed below represent the most commonly-used paradigms for studying binge eating in animals. Most models derived from binge-eating related disorders have characterized binge-eating and overeating behaviors, with limited models assessing associated symptoms such as self-induced vomiting (see e.g., sham-feeding model in [9]). In most paradigms, binge eating is measured as the presence of episodes where the animals eat large amounts of food in a short amount of time, beyond their homeostatic needs [10–12]. It is important to note that binge-like eating in animal models reflects the behavioral components, but not necessarily the emotional components of binge eating commonly observed in humans, such as distress and guilt [10].

In Table 1 we briefly evaluate major classes of common binge-eating animal models across three commonly invoked types of model validity (e.g., see [13]): face validity (similarity to binge-eating in humans), construct validity (alignment with the underlying theoretical concept), and predictive validity (relationship to the outcome of an experimental manipulation). Importantly, the relative importance of each type of validity depends on the specific research question (e.g., mechanistic studies vs. treatment prediction), a distinction often underemphasized in preclinical work.

**Table 1:** Evaluating model validity across animal models of binge eating.

Model type	Face validity	Construct validity	Predictive validity	Key references
Food restriction models	Moderate	High (restriction-binge cycle)	Moderate	[10, 100, 101]
Food addiction models	Variable	High (addictive processes to palatable foods)	Moderate/high	[23, 24, 51]
Stress-induced models	High	Moderate	Low/moderate	[56, 57, 61]
Conditioning models	Low/moderate	High (reward reinforcement)	High	[46, 90, 91]

### Food restriction models

Several animal models use various protocols of food restriction to induce binge eating. Clinically, the cycle of restriction and binging is a documented phenomenon (e.g., [14, 15]). In the limited-access model, rats are typically given access to shortening, a type of highly palatable fat commonly used in experimental diets, for a limited amount of time every day, while still having regular chow and water available at all other times [10]. A variant to this procedure is the intermittent access model, where the animals are given access to shortening intermittently on certain days [16]. Repeated fasting/refeeding (RF) cycles are often used to induce binge eating. One group (RF) engages in a 24 h fast followed by a 24 h refeeding period *ad libitum*, while the second group (control group) has free access to chow and water for the entire time [17]. Hyperphagia is then defined as increased food consumption following fasting compared to the control group. Some researchers have developed unpredictable access models, where access to palatable foods is given on random days at a random time, with the hopes of increasing face validity of induced binge eating [18]. Finally, the Binge eating resistant/Binge eating prone (BER/BEP) protocol developed by Boggiano (2007) is commonly used. In this model, a highly palatable food is introduced, and the rats are getting open access to it for 24 h, three times a week. Their access to foods is restrained except for regular meals the rest of the time. Rats who engage in excessive consumption of foods despite meeting their nutritional needs during the feeding tests are labeled as BEP, while the rest are labeled as BER.

### Food addiction models

Other models of binge eating are derived from addiction paradigms. Food addiction is a proposed behavioral addiction characterized by loss of control over eating, compulsive overeating, food obsessions, and an inability to stop overeating despite adverse consequences [19, 20]. It gained interest following the expansion of the definition of addiction in the DSM-5. Whether or not it is a valid diagnosis is still controversial, mainly because of its documented overlap with BED in humans [21, 22]. Animal models of food addiction use palatable foods (high-fat or high-sugar) to induce binges and other associated addictive-like behaviors in rodents, by limiting the availability of the foods. These models are similar to food restriction models, except that they measure behaviors specifically associated with addiction (e.g., tolerance, withdrawal-like behaviors, substance seeking). For instance, Avena and Hoebel (2006) developed a sugar binging model in which rats are experiencing 12-hour food deprivation, then have access to a 25% glucose or 10% sucrose solution for 12 h [23]. Over time, they increase consumption of the sucrose solution in a short amount of time and present potential signs of addiction (i.e., high motivation, withdrawal symptoms, anxiety). Similarly, de Jong and colleagues (2012) describe an animal model of food addiction based on a cocaine addiction model. Their model focuses on inducing and measuring three key behaviors: motivation for the substance, persistent substance seeking even when it is no longer available, and persistent substance seeking despite negative consequences [24]. Nevertheless, as Epstein and Shaham (2011) point out in a commentary, interpretations of such models should be made cautiously. They emphasize that “food addiction” in rodents is not equivalent to human obesity, and that, even in addiction contexts, factors like choice play an important role [25].

### Stress-induced models

Combining food restriction and stress has been shown to induce binge eating in rodents. In these models, acute stress, such as foot shocks, is combined with periods of food restriction. For example, in a model described in Boggiano (2006), rats are divided into four groups: one subjected to cyclic food restriction, one subjected to acute foot shock stress only, another subjected to both, and a control group. Eating behaviors are then measured and compared. Due to the reliance of this model on acute stress, an alternative model of emotional stress-induced binge eating was proposed by Anversa and colleagues (2020). In the latter model, mice are exposed to food restriction and frustration stress, where they can see and smell palatable food but do not have access to it [26]. A naturalistic model of chronic subordination stress using a social defeat model was also developed in mice [27], whereby subordinate animals that have a complex profile of behavioral and metabolic issues (e.g., depressive-like symptoms and up-regulated stress response system known as hypothalamic-pituitary-adrenal (HPA) axis) were shown to display spontaneous binge eating. The researchers argue that the phenotype can be representative of binge-eating-related disorders.

### Conditioning models

Operant conditioning procedures have also been used to study binge eating. In operant binge-like eating procedures, rats are trained to respond to food rewards under controlled conditions. The operant conditioning usually takes place over more than 15 days [28]. Key variables being manipulated are the reinforcement schedule, food type (e.g., sugar vs chow), and food availability (restricted access to foods at certain times). One variation that was recently developed includes intermittent access and unpredictability to assess motivation and cue control [29]. Once the behaviors stabilize, the rats are exposed to a progressive ratio of reinforcement for food. Control groups undergo similar procedures with chow instead of palatable foods. In these models, binge-like behavior is typically defined by lever-pressing rates, food intake, and eating patterns.

### Criticisms and limitations of existing animal models of binge eating

Key drawbacks of animal models should be acknowledged as we consider the clinical implications of their findings and possible future improvements. First, the models of binge eating described focus on quantifiable behavioral components of binge-eating-related disorders. To date, researchers are unable to model certain key ED symptoms present in those disorders, such as fear of gaining weight, subjective binges, calorie counting, the sense of lack of control in binging, self-induced purging behaviors, and body image issues [30]. The exclusion of such components elevates concerns over the validity of the models concerning human symptom presentation. Second, more broadly, there is an issue of oversimplification of binge-eating behaviors. Persistent interest is given to palatable foods and the addictive nature of fat and sugar, but that does not explain the variety of foods that are binged on. These models also do not account for the diversity of motives behind binge-eating behaviors (e.g., intrinsic versus extrinsic motivations). Third, food restriction is often used to induce binge eating in rodents. However, although food insecurity can increase risk for EDs, food restriction in human EDs is rarely induced by only external factors. Fourth, there is a general lack of consideration for ED diagnoses other than AN and BED in ED animal research. Specifically, little is still known about how to replicate the relation between binge eating and compensatory behaviors as they are observed in BN into animal models. Shifting diagnostic criteria and diagnostic crossover in humans – a common clinical reality – also introduces clinical heterogeneity that cannot be replicated in animals to the same extent. Fifth, research with animal models often focuses on the biological perspective when studying ED etiology. This translates into research objectives overly reliant on the weight and food consumption aspects of EDs, as can sometimes be seen in the food addiction literature. Finally, there is a need to be careful not to confound obesity and EDs, as the former is not considered a psychiatric disorder. It can be stigmatizing to automatically associate binge eating with obesity and poor health habits. There are similarities and differences between animal models used to study EDs and obesity (as highlighted in [31]), such as the presence of diet manipulation in both models. Although BED is common in individuals with obesity, it is not limited to this group [32]. Around 20 to 30% of individuals with BED do not have obesity [33, 34]. Similarly, key associated characteristics of binge eating, such as loss of control and emotion dysregulation, are differently associated with obesity [35]. For instance, loss of control over eating seems more strongly linked to psychological distress in individuals with BED than in individuals with obesity without BED [36, 37]. Still, in the context of BED specifically, it is important to acknowledge its associations with early onset of obesity and weight gain [31].

Collectively, the preceding limitations highlight the need for clearer translational goals and more explicit alignment between clinically-defined features of binge eating and the behaviors, mechanisms, and outcomes assessed in animal models. In the following section, we address possible key translational priorities.

## Translational priorities for modeling binge eating

Five translational priorities were derived from human clinical research and psychopathology models of binge eating, such as the transdiagnostic cognitive-behavioral model of EDs [7] and the biopsychosocial etiological model [4, 38, 39]. These were chosen based on the most studied themes within the animal binge-eating literature. Specifically, the themes of each of the priorities reflect core features of binge eating: (1) loss of control and compulsivity, (2) negative affect and stress responsivity, (3) developmental timing and sex differences, (4) individual differences and variability, and (5) treatment responsiveness. Together, these priorities provide a structure for assessing the strengths and limitations of current models, and for identifying feasible directions for refinement.

### Loss of control and compulsivity

Loss of control over eating is central to the clinical experience of binge eating and has been shown to be more strongly associated with distress and impairment than the actual size of the binge-eating episode [40]. Clinically, loss of control refers to the subjective inability to regulate eating behavior, regardless of the amount consumed [36, 41]. Preclinical studies have identified several processes that may contribute to loss of control, including heightened reward sensitivity, persistence despite adverse consequences, and neurobiological alterations.

Animal models have been used to examine behavioral and neurobiological processes that overlap with those implicated in substance use disorders [24, 28, 42, 43]. For instance, a sugar-bingeing model has been used to show that similar neurochemical changes can be observed when mice are bingeing on sugar, such as dopamine and acetylcholine release in the nucleus accumbens (NAc). These same rats also exhibit similar behaviors to drug addiction, such as enhanced intake following abstinence and cross-sensitization [42, 43]. Overlap in brain regions activated when rats binge on palatable foods has been documented in regions such as the NAc, orbitofrontal cortex (OFC), hippocampus (particularly, CA3 region), amygdala, and striatum (such as documented decreased dopamine D2 receptor availability). These regions are thought to be involved in both food and drug craving [24]. Taken together, these animal studies suggest addictive-like properties of certain foods and show common neurobiological substrates to substance abuse and binge-eating behaviors. Importantly, translating these findings into clinical frameworks remains challenging. Labelling certain foods as “addictive” could shift treatment approaches toward harm reduction strategies focused on avoidance or restriction, which contrasts with evidence-based eating disorder treatments that emphasize dietary flexibility and food variety [44, 45]. Incorporating addiction-specific psychoeducation and language into the treatment of EDs and global health campaigns targeting obesity could in certain cases reinforce restrictive dietary rules. This, in turn, may negatively affect the recovery of individuals presenting with EDs.

Reward and motivation processes are also thought to underlie clinical features adjacent to loss of control, such as eating when not physically hungry and despite negative consequences. For instance, one study illustrated a mouse model of binge-like sugar overconsumption, where mice developed binge-like excessive behavior, which persisted even when they consumed substantial chow beforehand, mimicking human “eating when not physically hungry” [46]. While the homeostatic pathway regulates the organism’s energy balance by increasing the motivation to eat in the event of energy store depletion, hedonic (or reward-based) regulation may override the homeostatic regulation by increasing the desire to consume highly palatable food, even in the context of energy abundance [47]. Feeding control and energy balance signals such as leptin, ghrelin, and melanocortin have been shown to interact with dopaminergic pathways in the nucleus accumbens and ventral tegmental area [48], suggesting they may modulate loss of control beyond metabolic need. Similarly, animal models have shown increased motivation to binge eat despite aversive consequences. Moshe and colleagues (2017) found that lactose-conditioned BEP rats exhibited heightened motivation towards palatable food despite aversive consequences, unlike BER rats, who devalued those foods over time due to overeating-related abdominal discomfort [49]. Furthermore, binge eating has been linked to alterations in key brain regions involved in reward processing and motivation. The NAc and medial prefrontal cortex (mPFC) seem to play crucial roles in assessing food palatability and driving reward-seeking behaviors, with increased sensitivity observed in BEP rats [50]. Involvement of the dopamine system has also been examined in animal models [51]. Heightened activity of dopamine D1 receptor-expressing neurons in the NAc and caudate putamen has been associated with binge eating [52]. Anticipatory activation of dopamine receptors in response to palatable foods further suggests altered reward processing [53]. Lastly, opioid system neuroadaptations in the mPFC may further contribute to binge-eating behaviors following intermittent access to highly palatable food [54].

Taken together, animal studies indicate that loss of control over eating reflects the interaction of heightened reward sensitivity, altered motivational processing, and impaired behavioral regulation. More attention should be given to modelling intrinsic motivation and loss of control beyond quantity of food intake [36, 41]. Indeed, potential ways of including these elements into binge-eating models could be through the inclusion of more varied behavioral outcomes, such as effort-based access.

### Negative affect and stress responsivity

Clinically, binge eating is often associated with negative affect and difficulties with emotion regulation, not necessarily direct stress exposure [55]. The impact of stress on binge eating has been studied in animals, as various stressors (e.g., childhood maltreatment, victimization) are considered relevant in the development of disordered eating [26, 27, 56, 57]. Specifically, there is evidence that stress can induce binge-like behaviors in rats, with the possible involvement of the HPA-axis. Combining dieting and stress has been found to reproduce key human characteristics of binge eating, such as a preference for palatable food and eating for reward rather than for homeostatic needs [56]. One study used a BEP model to show that stress-induced binge eating may be mediated by increased motivation to eat palatable foods despite consequences, and a dysregulated HPA axis [57]. Building on this, Di Bonaventura and colleagues (2014) showed that frustration stress (induced by preventing access to a visible palatable food) can trigger binge eating in food-restricted female rats, an effect mediated by corticotropin-releasing factor receptors in the bed nucleus of the stria terminalis [58]. With the goal to enhance validity, some researchers have attempted to diversify paradigms of stress. For instance, Anversa and colleagues (2020) focused on inducing binge eating through emotional stress rather than physical stress in female mice. They concluded that binge eating could be induced without food restriction and was more present in females than males [26]. Neuroendocrine signals involved in both stress and appetite regulation may further shape binge eating that is driven by emotions, as interactions between the HPA axis and ghrelin signaling have been shown to influence stress-induced feeding in animal models [59, 60].

Although stress manipulations reliably modulate binge-like intake, they do not necessarily capture the internal states that characterize emotion-driven binge (for review, see 59). Incorporating measures of affective reactivity, individual differences in stress sensitivity, or repeated stress/binge cycles may better capture clinically relevant binge eating. Additionally, attempts to incorporate key emotional variables into binge-eating animal models suggest that anxiety and depression could be considered more often as indicative of BEP rats [61]. Models that disentangle stress exposure from affective regulation processes may better capture clinically relevant pathways to binge eating.

### Developmental timing and sex differences

From a clinical standpoint, binge eating commonly emerges during adolescence (for review, see [16] and [20]) and shows sex differences in prevalence [5], suggesting that developmental timing and hormonal context are core features rather than secondary variables. Animal research has contributed to our understanding of developmental factors linked to binge-eating symptoms [62–65]. A few studies have examined the associations between puberty and binge-eating behaviors. For instance, a longitudinal study by Klump and colleagues (2011) using a BEP/BER model has found that female rats in mid-late puberty were more likely to binge eat than female rats in pre- or early puberty. Maternal separation has also been examined as a possible contributor to binge-eating behaviors. Ryu and colleagues (2008) found that rats who had undergone maternal separation following birth presented with persistent binge eating and alterations in HPA axis functioning compared to those who had not. Quality of the environmental stimulation in developing rats has been suggested to impact feeding behaviors; a limited access model showed that an enriched environment delayed the first binge episode, but did not alter the amount of fat consumed [64]. More recently, exposure to palatable foods during adolescence has been shown to be associated with increased binge eating, with hormone fluctuations as a possible mediator [66]. Developmental changes in metabolic and appetite-regulating hormones during puberty, such as fluctuations in leptin and ghrelin [67], may further shape vulnerability to binge eating, although this association remains unclear.

Sex differences also emerge as a prevalent focus of the animal literature. Several studies highlight factors that could potentially explain binge-eating proneness in females [68–73]. Female rats are often used in binge-eating animal models due to their higher propensity for these behaviors in both rats and humans. Still, there seems to be an increasing inclusion of male rats in binge-eating animal research, especially in studies focusing on sex differences. For example, there is evidence that palatable foods may be more rewarding to females through a heightened response of neural substrates involved in motivation [73]. In the BEP/BER rodent model, females are consistently two to six times more likely to present with the BEP phenotype [70]. Certain hormones also seem to be regulating food intake differently in males and females; ghrelin, a hormone that stimulates hunger and reward signaling, when deficient, was found to be associated with lower food intake in female rats [72]. The effect of 2-hydroxyestradiol (2-HE2), a metabolite of the major female sex hormone estradiol, on binge eating in female and male rats is being explored. One study showed its influence on emotional binge eating; ovariectomized rats, when untreated with 2-HE2 and subjected to food restriction, displayed binge eating and had increased cell expression in the amygdala, hypothalamus, and the stria terminalis [71]. Two studies found that 2-HE2 supplementation amplified binge eating in female rats, and that it suppressed dopamine signaling in the prefrontal cortex [68, 69].

Despite the clinical relevance of adolescence and sex differences in binge eating, many animal studies rely on adult, single-sex subjects, limiting generalizability to high-risk populations and meaningful comparisons. Systematic incorporation of developmental timing and sex as design variables, rather than post-hoc comparisons, would strengthen translational relevance and improve alignment with epidemiological research.

### Individual differences and vulnerability

Clinically, binge eating is characterized by marked heterogeneity in clinical characteristics (severity, course, and comorbidity) and vulnerability factors. Animal research has significantly advanced our understanding of genetic vulnerabilities related to binge eating disorders [74–78]. Firstly, several gene expression alterations associated with binge eating have been identified. For example, the expression of the A2aAR and GHSR genes, responsible for activating the adenosine A2A receptor and the ghrelin receptor, respectively, appears to influence food consumption [77, 79]. Additionally, changes in the expression of other genes, including the dopamine D2 receptor gene and the serotonin receptor 2 C gene, within the CA3-OFC-NAc neural network, have been linked to deficits in contextual and reversal learning in binge-eating rodents [75]. Furthermore, studies have identified two strains of mice associated with binge eating. The 129S1/SvlmJ strain has been linked to reduced binge eating [78], while the C57BL/6NJ strain is associated with increased binge-like consumption of sweetened, palatable food [74]. In summary, these findings highlight the potential of genetic research in identifying genes and substrains associated with binge eating.

While genetic and strain-based approaches have highlighted vulnerability factors for binge eating, they are often studied in isolation from environmental and developmental influences. Future work integrating genetic susceptibility with stress exposure or developmental timing would more closely align with multifactorial risk models from human research. Additionally, more research has been coming out highlighting the role of epigenetic processes in understanding ED etiology [80, 81]. It would be worth expanding this area of research for binge eating-related disorders specifically, using animal models.

### Treatment responsiveness

In a clinical context, effective treatment of binge eating is typically defined not solely by reductions in number of binge-eating episodes, but by improvements in loss of control, quality of life, associated distress, and relapse vulnerability. Animal models offer an opportunity to examine neurobiological and behavioral mechanisms underlying treatment sensitivity under controlled conditions, and to identify candidate targets that may inform clinical intervention.

Animal research has elucidated various neural and pharmacological mechanisms underlying binge-eating behavior and has identified potential therapeutic targets. Namely, nociception receptor signaling [82] and n-methyl-d-aspartate (NMDA; [83]) receptors appear to regulate binge eating of palatable foods, thereby presenting an attractive target for ED treatments. Similarly, cannabinoid receptors have been identified as maintaining excessive intake in sweet-fat diet binges [84]. Additionally, dieting history has been found to affect serotonin function, impacting feeding and brain-based reward systems via central dopamine/serotonin interactions [85].

Some animal studies attempt to reduce binge-eating behaviors through brain stimulation, a potential treatment avenue. For instance, high-frequency deep brain stimulation (DBS) of the NAc core has been found to reduce binge-eating episodes in both a chronic binge-eating model, where intermittent access was used to induce binge eating, and in a relapse model, where rats were re-exposed to palatable food following abstinence [86]. Similarly, low-frequency stimulation of the prelimbic cortex, a region of the mPFC known for its involvement in substance seeking and craving, significantly reduces binge size, suggesting the implication of prefrontal hypofunction in binge eating [87].

In terms of direct interventions for binge eating, animal models have been used to test the effect of potential compounds (see Table 2 for a summary). Adenosine A_2A_ receptor agonist CGS 21680 was found to inhibit both high-palatable food intake in sated rats and low-palatable food intake in food-deprived rats, highlighting their potential utility in controlling binge-related eating disorders and obesity [88]. Likewise, N-acetylcysteine (NAC), the mainstay for therapy of psychostimulant (i.e., acetaminophen) toxicity, was shown to reduce binge eating of a Western diet without affecting standard chow consumption [89]. The NMDA receptor antagonist memantine was found to decrease binge-like eating and food-seeking behavior in palatable food groups without affecting control groups, indicating its potential as a treatment for binge eating [90]. Lisdexamfetamine (Vyvanse) reduces chocolate bingeing through indirect α1-adrenoceptor and possibly dopamine D1 receptor activation [91]. Lastly, glucagon-like peptide-1 (GLP-1) receptor agonists (e.g., liraglutide, semaglutide) have become the focus of recent attention as a potential pharmacological treatment for binge eating and obesity. GLP-1 receptor signaling in the VTA and NAc has been associated with the regulation of food intake in rats [92, 93]. This compound (GLP-1RA), originally developed as a treatment for type 2 diabetes, has been found to reduce binge-like eating in rats [94, 95].

**Table 2:** Summary of compounds tested with animal models of binge eating.

Compound	Market name	Mechanism of action	Summary of outcomes
CGS 21680 [88]	N/A	Activation of A_2_A receptors and stimulation of adenylate cyclase, modulates dopaminergic and immune functions.	Found to inhibit palatable and non-palatable food intake.
N-acetylcysteine [89]	Mucomyst, Acetadote	Modulates glutamatergic system via cystine-glutamate antiporter.	Found to reduce binge-eating behaviors.
Memantine [90]	Namenda	Non-competitive NMDA receptor antagonist regulates glutamatergic neurotransmission.	Found to decrease binge-eating behaviors and food-seeking behaviors of palatable foods.
Lisdexamfetamine [91]	Vyvanse	Metabolizes into dextroamphetamine, impacts release and reuptake of monoamines.	Found to reduce binge eating of palatable foods.
GLP-1 receptor agonists [92–95]	Semaglutide (Ozempic), Liraglutide (Victoza), and more.	Glucagon-like peptide-1 receptor agonists, impacts glucose metabolism and appetite regulation.	Found to reduce binge-eating behaviors.

Beyond preclinical animal models, drugs targeting certain receptors and transporters, including (but not limited to) dopamine transporter and receptors, and NMDA receptors, appear to reduce symptoms of binge eating in humans [96, 97]. That being said, GLP-1 receptor agonists have been found to have potential negative consequences in humans, such as pancreatitis, acute kidney injury, thyroid C-cell tumors (found in rodents), and more [98, 99]. The use of this type of medical treatment should thus come with careful indications of use and awareness of potential risks. At this moment, they are not considered to have sufficient data to be recommended for treatment for binge eating specifically (for review, see 99). Further research in animals and in humans is needed to maximize efficacy and reduce the risks of such medical treatment in patients.

Many animal models demonstrate sensitivity to pharmacological manipulations, yet the extent to which these responses predict clinical efficacy remains inconsistent. Aligning outcome measures with clinically-meaningful endpoints, such as reductions in loss of control or relapse behavior, may enhance predictive validity and improve the translational utility of preclinical treatment studies.

#### Synthesis of translational recommendations

Taken together, the translational priorities outlined above highlight recurring gaps between clinically-defined features of binge eating and their current operationalization in animal models. Several studies published in the past decade have already proposed targeted refinements to binge-eating paradigms that align closely with the translational priorities outlined above [18, 61, 100–104]. Some have criticized commonly used food access conditions, highlighting the need to account for the unpredictability of human binge-eating behaviors in mice models of binge eating, showing that unpredictable access to palatable foods can induce binge eating [18, 104]. The length of access to palatable foods continues to be challenged, with short- and long-term access leading to different phenotypes of binge-eating behaviors. Long access has more similarities with human BED and should be considered in future studies [101].

Table 3 synthesizes the five priorities by mapping the key clinical features of binge eating onto commonly-used animal models, identifying specific gaps, and outlining feasible strategies for refinement. This framework is intended as a practical guide for preclinical researchers seeking to enhance clinical relevance without abandoning established paradigms, and for clinically-oriented readers aiming to interpret the translational strengths and limitations of existing models.

**Table 3:** Synthesis of translational recommendations.

Translational priority	Common limitation	Proposed refinement/ recommendation	Example paradigms or approaches	Translational value
Loss of control and compulsivity	Intake magnitude used as primary outcome; nature of the engagement and conceptualization unclear	Incorporate effort-based access, or other varied behavioral outcomes reflecting loss of control (e.g., persistence despite consequences)	BEP/BER models; conditioned aversion paradigms; progressive ratio tasks [34, 35]	Improves construct validity for loss of control, independent of intake
Negative affect and stress responsivity	Stress treated as an acute trigger rather than an affective vulnerability	Use repeated or emotional stressors; assess affective reactivity and stress-binge cycles	Emotional stress paradigms; frustration stress models [15, 63, 93]	Improves face validity, emotion-driven binge eating observed clinically
Developmental timing and sex differences	Reliance on single-sex subjects; developmental variables neglected	Systematic inclusion of adolescence and hormonal state as design variables	Puberty-focused models; hormone manipulation studies [20, 52, 53]	Aligns models with high-risk developmental windows
Individual differences and vulnerability	Homogeneous samples; limited modeling of resilience factors	Use strain differences or selective vulnerability approaches	BEP/BER classification; strain comparisons [42–45, 47]	Captures heterogeneity seen in clinical populations
Treatment responsiveness	Treatment effects assessed primarily via intake reduction	Evaluate effects on loss of control, relapse-like behavior, or compulsivity	Pharmacological and DBS models with behavioral endpoints [70, 71, 74]	Enhances predictive validity for clinical efficacy

Advancing translational relevance will require linking the behavioral phenotypes modeled in animals to neurobiological markers that are meaningful at the clinical level. Animal models of binge eating have identified several relevant neurobiological alterations, including dysregulation of the dopaminergic reward system [34–36], HPA axis hyperactivation linked to stress responsivity [55, 58], and neurotransmitter imbalances involving serotonin (e.g., [74]). Additionally, evidence from both animal and emerging human studies points to increased neuroinflammation (e.g., [94]), structural and functional brain changes [105], shifts in gene expression (e.g., [40, 42]), and specific gene polymorphisms [106]. However, the extent to which these markers reliably map onto clinical symptom severity, loss of control, or treatment response remains unclear. Integrative approaches combining behavioral assays, biological measures, and longitudinal designs will be critical for identifying mechanisms that translate across species and inform intervention development.

## Conclusions

In this review, we synthesized the existing literature by reorganizing animal models of binge eating around five translational priorities derived from clinical research: loss of control and compulsivity, negative affect and stress responsivity, developmental timing and sex differences, individual vulnerability, and treatment responsiveness. We highlight the value of animal models in examining key biological and behavioral mechanisms implicated in binge-eating-related disorders, particularly in the domains of reward processing, stress responsivity, and genetic vulnerability. Yet, we also underscore the need for better alignment between animal and clinical models. Current models rarely capture key features of binge eating, such as loss of control or emotional distress. Building on insights from clinical human research, we propose that refining animal paradigms to include more nuanced emotional and behavioral markers that reflect symptom heterogeneity, such as affective dysregulation, and clinically-meaningful treatment outcomes will enhance their translational utility. Ultimately, closer integration between animal and clinical research will be key to identifying meaningful treatment targets and developing more effective interventions for binge eating.
